# Is a Patient with Paget’s Disease of Bone Suitable for Living Kidney Donation?—Decision-Making in Lack of Clinical Evidence

**DOI:** 10.3390/jcm11061485

**Published:** 2022-03-09

**Authors:** Paweł Poznański, Agnieszka Lepiesza, Diana Jędrzejuk, Oktawia Mazanowska, Marek Bolanowski, Magdalena Krajewska, Dorota Kamińska

**Affiliations:** 1 Department of Nephrology and Transplantation Medicine, Wroclaw Medical University, Borowska 213, 50-556 Wroclaw, Poland; pawel.poznanski@umw.edu.pl (P.P.); oktawia.mazanowska@umw.edu.pl (O.M.); magdalena.krajewska@umw.edu.pl (M.K.); dorota.kaminska@umw.edu.pl (D.K.); 2 Department of Vascular, General and Transplantation Surgery, Jan Mikulicz-Radecki University Clinical Hospital, Borowska 213, 50-556 Wroclaw, Poland; alepiesza@usk.wroc.pl; 3 Department of Endocrinology, Diabetes and Isotope Therapy, Wroclaw Medical University, Wybrzeze L. Pasteura 4, 50-367 Wroclaw, Poland; marek.bolanowski@umw.edu.pl

**Keywords:** living donor kidney transplantation, living kidney donor candidate, guidelines, Paget’s disease of bone, bisphosphonates

## Abstract

Living donor kidney transplantation is a widely performed medical procedure. Living kidney donation requires an in-depth health assessment of candidates. The potential living kidney donor must remain healthy after kidney removal. A consequence of donation can be a decrease in glomerular filtration rate (GFR), and donors can become at risk of developing chronic kidney disease (CKD). We present a rationale for potential living kidney donor withdrawal due to Paget’s disease of bone (PDB) based on a literature review. The treatment for PDB includes the use of, for example, non-steroidal anti-inflammatory drugs (NSAIDs), which can lead to acute kidney injury (AKI) as well as CKD, or bisphosphonates, which are not recommended for patients with decreased GFR.

## 1. Introduction

Living donor kidney transplantation is a well-established and widely performed medical procedure. Due to its multiple advantages over deceased donor kidney transplantation, living donation should be a preferred source of kidney grafts. Despite its benefits (i.e., better and more sustained graft function), in some regions (including Poland), the bulk of organ procurement is still based on deceased donors. Living kidney donation requires an in-depth health assessment of candidates. The potential living kidney donor is, in principle, a generally healthy person, and must remain so, not only immediately after kidney removal, but also for the rest of their life. In this context, demanding health requirements for potential living donors are included in both the local and international guidelines for the evaluation of living kidney donor candidates who must meet the legal terms and conditions set forth in local and international legal acts (in Poland, the Polish Transplantation Act) [1,2,3].

## 2. General Approach to Living Kidney Donation Candidates

As mentioned previously, potential living kidney donors must meet conditions required by legal terms and guidelines formulated by scientific societies and foundations [1,2,4,5,6,7,8,9,10,11]. It should be noted that these are only boundary criteria and cover only typical factors such as the donor’s age, kidney function, hypertension, body mass index (BMI), diabetes, proteinuria, hematuria, nephrolithiasis (NL), and malignancies [12].

### 2.1. Balancing Donor Risks with Recipient Benefits

Living donor transplantation is obviously beneficial for both donors and recipients. The ethical principles should balance utility–justice and personal autonomy. Because the survival rate of kidney transplant recipients is much higher than that of waiting list patients, living donor transplantation, even during the COVID-19 pandemic, is categorized as tier 3b, which means that it should not be postponed [13]. The risk exposure and outcome of transplantation depend on multiple factors and donor/recipient pairs should be engaged in a discussion of their expectations. Low rates of deceased kidney donation lead to increasing acceptance of so-called marginal kidney donors. This includes donors with diabetes, hypertension, elderly donors, and those with obesity as well as borderline eGFR. Two papers published in 2014 showed that all-cause mortality in patients after the donation was more than ten times higher compared to healthy nondonors [14,15]. However, a recent paper by Kinoshita showed that clinical outcomes of donation in medically complex living kidney donors did not adversely affect their renal health (but were associated with worse graft survival) [16]. Most living donor programs accept different levels of pathologies such as hypertension, obesity, or hyperglycemia stratified by donor’s age. Bearing in mind that living donor transplantation not only improves the recipient’s quality of life but also expands their lifespan—a donor-oriented approach should prevail [17,18]. In some countries including Poland, the applicable legal regulations are very restrictive. According to the Polish Transplantation Act, in the Polish legal doctrine, there is a discussion on the admissibility of organ transplantation ex vivo as subsidiary to donation ex mortuo (i.e., a living donation is possible only if an organ from a deceased donor is not available to the recipient). Nevertheless, this position is not fully grounded in the doctrine [19,20].

### 2.2. Kidney Function Requirements in Living Kidney Donors

One of the most crucial considerations is the glomerular filtration rate (GFR). Based on the most recent guidelines of Kidney Disease: Improving Global Outcomes (KDIGO Clinical Practice Guideline on the Evaluation and Care of Living Kidney Donors 2017) [2], kidney function can be assessed as sufficient for kidney donation (if eGFR is 90 mL/min per 1.73 m^2^ or greater) or insufficient for donation (if GFR is less than 60 mL/min per 1.73 m^2^). A result between these two values demands individualization and must comply with the scope of the transplant program and be within the limits of the acceptable risk. In the Living Donor Transplant Program in Wroclaw, Poland, in such situations, we make use of the British Transplantation Society and The Renal Association guidelines (BTS/RA Living Donor Kidney Transplantation Guidelines 2018) [11]. These guidelines are gender-specific and age-adjusted and help to predict whether maintained post-donation GFR will remain above the lower limit (−2 SD below mean) of the age and gender-specific normal range in association with pre- and post-donation end-stage renal disease (ESRD) risk predictor tools/calculators [21,22,23] (depicted in Table 1).

**Figure 1:**
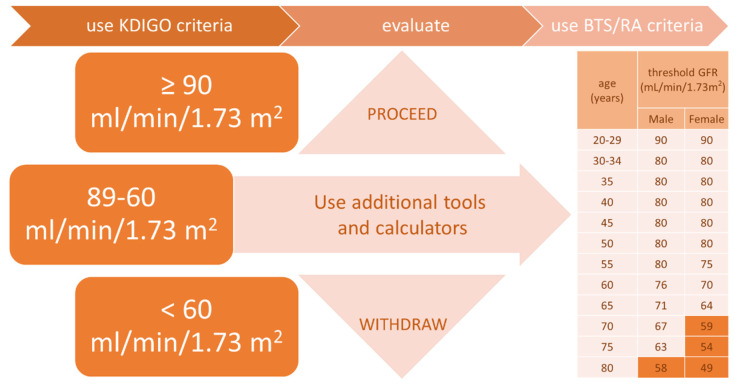
The decision-making flowchart for assessing pre-donation kidney function in a living donor. Conditions subsequently affecting kidneys (e.g., hypertension, diabetes, autoimmune disorders) should be excluded in further evaluation irrespective of the value of the acceptable eGFR (even above 90 mL/min/1.73 m^2^).

For these calculations, pre-donation variables such as age, gender, race, donor–recipient relation, diabetes in the donor, diabetes in the recipient, eGFR, blood pressure, hypertension medication, BMI, smoking, urine albumin/creatinine ratio, and glucose are used. The decision-making flowchart for the assessment of kidney function in a living donor is shown in Figure 1.

### 2.3. Kidney Function Assessment in Living Kidney Donors

As mentioned previously, the keystone of living kidney donation candidate evaluation is to assess the adequacy of renal function. Previously, it was thought that the risk of ESRD in the donor is not higher in comparison to the general population [24] or even that donors live longer [25]. However, according to the current literature, living donors should be assumed to be at risk of ESRD. Therefore, an accurate assessment of kidney function is crucial [14,15]. It is well described in the literature that unilateral nephrectomy with a compensatory hyperfiltration in the remaining kidney reduces GFR by approximately 30% in a one-year post-donation period [24,26] with further physiological, age-related reduction [27]. According to the KDIGO [2] and BTS/RA [11], all guidelines emphasize the importance of two-step evaluation with a screening test based on estimated GFR (eGFR) with an accurate equation (i.e., Chronic Kidney Disease Epidemiology Collaboration (CKD-EPI) creatinine equation) and confirmatory test with measured GFR (mGFR) using the exogenous filtration marker of renal clearance of 51Cr-EDTA or iothalamate and plasma clearance of 51Cr-EDTA or iohexol as sufficient methods [28].

However, other authors suggest that estimation of GFR with the use of equations consisting of variables such as serum creatinine, age, sex, body mass (i.e., Cockcroft–Gault, Bjornsson, Hull, and Mawer equations) correlates with pre-donation 24-h urine creatinine clearance and renal cortex volume measured using computed tomography (CT) [29,30]. Nevertheless, eGFR still appears insufficient compared to exogenous filtration markers [31] whose selection, if they are used, should be individualized and the evaluation should be based on more than one equation [32].

### 2.4. Beyond-the-Guidelines Requirements for Living Kidney Donation Candidates

**Figure 2:**
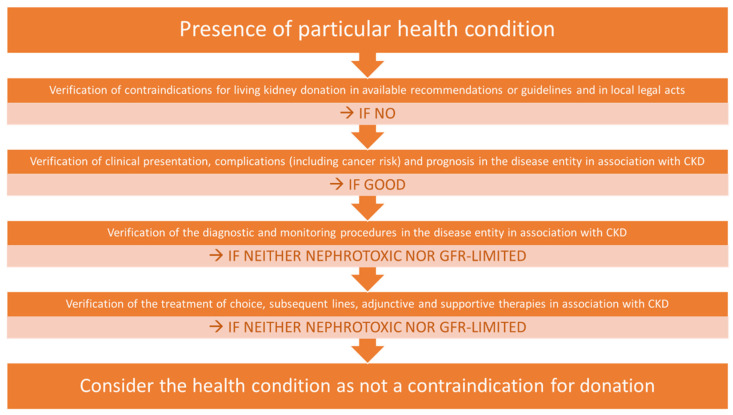
The decision-making flowchart for the living donor suitability assessment. Each step contains two possible answers. Continue if the assumption from every second (brighter) line is met.

Since it is pointless and impractical to establish qualification criteria for every known disease, determining contraindications for a specific health condition is the task of the local Living Kidney Donation Program Qualification Board to be carried out in cooperation with certain medical experts and the patient, and is based on the evaluation of the current scientific reports and literature review. This approach is in line with the paradigm of shared decision making and takes into account the risks, benefits, and the will of candidates for donors and recipients [33]. While the literature review revealed neither scientific reports of threats nor contraindications for donation in a particular disease, the proposed practice algorithm used at the authors’ center is presented in Figure 2.

### 2.5. The Real-World Application of the Algorithm Used for Infrequent Pathologies in Potential Living Donors

In clinical practice, it may occur that in classic donor evaluation procedures, poorly expressed or infrequent conditions can be easily overlooked. On the other hand, diagnosing an infrequent condition or a rare disease results in the necessity of organ procurement decision-making in the absence of, or only little evidence-based medicine (EBM). From our daily practice, we have evaluated potential living donors with a variety of occult pathologies, among others Birt–Hogg–Dubé syndrome, alpha-1 antitrypsin deficiency, Langerhans cell histiocytosis, Cacchi–Ricci disease, pneumatosis intestinalis, and Paget’s disease of bones. Herein, we present our practical algorithm used in real-life conditions encountered in our living donor transplant center, based on the case of a potential living donor with Paget’s disease of bone.

## 3. The Clinical Approach Illustration

### 3.1. The Clinical Presentation of Paget’s Disease of Bone (PDB)

PDB results from increased bone resorption due to hyperactivity of osteoclasts followed by the formation of bone by osteoblasts. PDB belongs to the group of chronic skeletal diseases with a focal manifestation with a single (monostotic form) or multiple (polyostotic form) localization. According to the clinical presentation, PDB may be asymptomatic or symptomatic. The frequency of medical appointments for patients with bone pain due to PDB varies, and depends on the analyzed area where PDB is more or less common. Previously, it was reported that 95% of PDB patients are asymptomatic [34], but more recent studies have shown that pain in the affected site is the most common symptom (41.6–78.3%) [35,36,37,38]. In their systematic review, Tan and Ralston noted that bone pain was the most common presenting feature (52.2% of cases), followed by deformity (21.5%), deafness (8.9%), and fracture (8.5%). Time trend analysis in subjects of European descent showed that fracture was less common in studies performed during the past 25 years compared with older studies (5.5 vs. 10.8%, *p* < 0.001) whereas pain was more common (54.3 vs. 48.3%, *p* = 0.003) [38]. In patients with PDB, the exacerbation of pain is one of the most reported symptoms that has a significant impact on functioning and quality of life [39]. Although bone pain is commonly reported in PDB patients because of impaired bone remodeling, disorganization of the bone architecture and microfractures due to mechanical stress, pain could also result from the involvement of nerves in bone with consequent development of peripheral and central neuropathic pain.

PDB is more common in males. Worldwide prevalence is diverse and could possibly depend on local/regional factors as well as other unrecognized ones [40]. In the United Kingdom, the prevalence of PDB in individuals older than 55 years reaches 2%. The incidence of PDB has declined rapidly in recent years [41]. In Poland, PDB is infrequently diagnosed and the epidemiological data are scarce but suggestive of a declining incidence [42].

### 3.2. Risks Associated with Living Kidney Donation in the Context of PDB

PDB is associated with several possible complications potentially requiring treatment. They include musculoskeletal, neurological, and cardiovascular system complications as well as metabolic disorders, neoplasms, and side effects of treatment [43]. In this setting, living kidney donation may raise several concerns. The overarching consideration is the potential necessity to preserve the function of the remaining kidney. Indeed, a consequence of donation is a decrease in kidney filtration function after kidney removal [44]. Furthermore, donors are at a small, but increased risk (compared to non-donors) of developing advanced chronic kidney disease (CKD) [45].

### 3.3. PDB and Neoplastic Risk

PDB carries an increased risk of malignancies such as sarcomas (up to 1%) and benign but aggressive non-cancerous giant cell tumors [43]. This is of concern as antineoplastic agents may have nephrotoxic side effects, especially when GFR is reduced. The mechanism of nephrotoxicity varies between agents in terms of affecting glomeruli, tubulointerstitial compartment, and microvasculature, with the clinical presentation ranging from a mild elevation of serum creatinine to advanced acute kidney injury requiring renal replacement therapy [46].

### 3.4. Metabolic Complication and CKD Overlap

Metabolic complications of PDB include hypercalcemia, hypercalciuria, hyperoxaluria, and hyperuricemia [43]. Disturbances in calcium homeostasis can lead to kidney stone formation and nephrolithiasis (NL), the most frequent kidney complication of PDB [47]. NL has been described as a possible non-malignant metabolic complication of PDB [43]. In the literature, a diagnosis of PDB in a patient during evaluation for NL was described, with pain in the lumbar region as the first symptom [48]. The patient had severe bilateral NL with gross pelvicalyceal dilatation of the right kidney and right hydroureter. Renal pyelogram and renogram showed almost non-functioning right kidney (GFR of 10 mL/min) with multiple left-sided renal calculi (GFR of 55 mL/min). Hyperparathyroidism (HPTH) was excluded, the findings of a bone scan were typical for PDB and despite the normal activity of alkaline phosphatase (ALP), after a bone biopsy, the diagnosis of PDB was confirmed [48]. The incidence of NL in patients with PDB without HPTH was estimated at 16%, while in the control group (without PDB and HTPH) it was 8%. This association remained significant also in a model adjusted for age, sex, BMI, and eGFR [OR 2.26 95% C.I.: 1.72–2.98, *p* < 0.01] [47]. The rate of recurrence of NL was higher in the polyostotic group than in the monostotic group of patients with PDB. These studies were performed on a group of PDB patients in metabolic remission (normal ALP activity), indicating that PDB itself is an independent risk factor for NL [47].

Mineral-bone disorder (CKD-MBD) is frequently diagnosed in advanced chronic kidney disease and associated with typical laboratory findings such as abnormal metabolism of calcium (hypocalcemia) and phosphate (hyperphosphatemia) and increased iPTH concentration. CKD-MBD leads to abnormalities in bone turnover, mineralization, and vascular or extraosseous soft tissue calcifications [49]. Patients with CKD-MBD compounded with PDB-related hypercalcemia could be at increased risk of NL. Moreover, increased serum phosphorus levels and calcium-phosphorus product concentrations are considered as risk factors for coronary artery disease, especially in predisposed individuals [50]. Additionally, studies have revealed that living kidney donors develop some abnormalities typical for CKD-MBD, even if only a mild reduction of GFR occurs [51]. Recent papers have shown that living kidney donors presented a significant change in mineral bone metabolism after donation, especially if preexisting comorbidities such as hypertension were present. Mineral bone metabolism abnormalities were more prevalent in donors with a greater relative decline in GFR after donation [52]. Another study showed early biochemical changes after donation compatible with CKD-MBD [53]. It was not shown that these metabolic changes may influence bone mineral density or increase fracture rates [54].

### 3.5. Further Diagnostics and Monitoring of PDB and CKD

Bone scintigraphy is the most sensitive method of detecting PDB and assessing whether the disease is monostotic or polyostotic [55]. Unfortunately, the bone scan does not seem to be specific, but there are some very characteristic signs that help diagnose PDB (the “Mickey Mouse” sign in the spine, increased radiotracer uptake diffusely in the proximal or distal part of a long bone with a sharp margin) [55,56]. X-rays are also effective because the radiographic features include cortical thickening, sclerotic changes, and osteolytic areas [57]. However, although the recent studies do not fully support the previous observation for nephrotoxicity of contrast media used during MRI and CT, there are still limitations on using contrast-enhanced MRI and CT in association with CKD [58,59,60,61,62]. Clinical and practice guidelines for the diagnosis of PDB apply to CT and MRI examinations only in some cases of complications and in the presence of neoplasms; in other cases, radionuclide bone scans and X-rays are sufficient [43,63]. Due to the emerging complications of PDB, it should be taken for granted that tests with the use of an X-ray (including computed tomography) will be necessary. The complication rate in PDB is rather high (52.2%) [36]. The most important, but rather uncommon complication of PDB is osteosarcoma. Osteosarcoma is one of the most common primary tumors of bone, with a 5-year survival rate of less than 20% after the development of metastases. Patients with Paget’s disease of bone are highly predisposed to osteosarcoma, and both diseases have common characteristic skeletal features due to rapid bone remodeling. Paget sarcoma is a particularly aggressive malignancy arising within a Pagetic bone, associated with a worse outcome than conventional osteosarcoma. A late peak is seen after the age of 50 years (peaking at 70 years). In a Tunisian report, the frequency of occurrence of osteosarcoma was 1.4% [36]. The historical data showed that histological analysis in regions of high PDB prevalence has revealed that 50% of osteosarcoma patients > 60 years of age have underlying PDB [64]. Other major complications of PDB include osteoarthritis (23.2%), followed by deafness (17.4%), fractures (15.9%), hydrocephalus (7.2%), and neurological diseases (7.2%) [36]. Pagetic patients often undergo orthopedic/neurosurgical procedures such as total hip or/and knee replacement, femoral and tibial osteotomy, correction of spinal stenosis or nerve root compression, vertebroplasty for painful vertebrae, ventricular-peritoneal shunting for hydrocephalus, suboccipital craniectomy and cervical laminectomy for basilar impression [65]. Almost all of the complications and their treatment listed above are related to the increase in the number of tests performed with gamma radiation.

### 3.6. CKD as an Armamentarium-Limiting Factor in PDB

The type of treatment approach to PDB depends on the disease presentation. The decision of whether to treat or not depends on the presence of symptoms. In symptomatic PDB, the treatment of choice includes antiPagetic agents as well as adjunctive therapies. In asymptomatic PDB, the introduction of treatment depends on the localization of the Pagetic bone and evidence of disease activity and is based on antiPagetic agents with a bisphosphonate (BP)—predominantly zoledronic acid [43,63,66,67]. The mechanism of action includes inhibition of enzymes involved in bone resorption by osteoclasts. In PDB, bisphosphonates (BPs) are used not only as anti-resorptive agents, but also as bone turnover-decreasing and analgesic drugs. Although other drugs such as analgesics, nonsteroidal anti-inflammatory drugs, and anti-neuropathic agents are often used in the management of bone pain associated with PDB, these agents have not been investigated in controlled clinical trials.

Recent studies indicate a high effectiveness of zoledronic acid in relieving pain and causing a significant decline in ALP in comparison to other BPs [68]. A single dose of 5 mg zoledronic acid was more effective in pain relief than 30 mg risedronate sodium daily [63]. A total of 88% of patients treated with a single dose of 5 mg zoledronic acid intravenously achieved a sustained and stable normal ALP level during the 5-year follow-up—much more than the 47% reported for oral risedronate sodium [69]. Another study showed that a single intravenous infusion of 4 mg zoledronic acid was more likely to relieve pain than 30 mg intravenous pamidronate when administered on two consecutive days every three months [70]. However, there was no difference in bone pain when comparing intravenous administration of pamidronate 60 mg every three months with oral alendronic acid 40 mg daily in 3-month blocks [71]. It has been suggested that an appropriate indication for BP treatment in PDB is to control bone pain thought to be due to disease activity [72]. Moreover, recent guidelines on PDB recommend treatment only for pain relief [63], while there is no evidence of its benefit in preventing disease complications in asymptomatic patients [66]. However, some patients do not respond to a single infusion of zoledronate or achieve only a transient disease control with the need of retreatment [73].

At this point, it is necessary to mention severe bone complications, which paradoxically may occur during the long-term use of BPs, which include atypical femoral fractures (AFFs) and bisphosphonate-associated osteonecrosis of the jaw (BONJ). AFFs are transverse sub-trochanteric fractures that occur after minimal trauma or in the absence of trauma. The exact mechanism of AFFs associated with BP use still remains unknown. Human and animal histological studies propose a process that mimics a stress fracture due to impaired bone healing and reduced osteoblast and osteocyte activity. In addition to the surgery of AFFs, the pharmacological treatment includes parathormone (PTH) analog (teriparatide) administration. On the other hand, the use of BPs after orthopedic surgery with endoprosthesis implantation (as a treatment for the consequences of PDB) can improve fixation stability at the bone–implant interface. The effects of BPs either reduce the time to union or enhance an impaired healing process [74]. Another severe complication of I.V. BP treatment, with the ranges of incidence between 5% and 20%, is osteonecrosis of the jaw (BONJ). Similar to AFF, it can be a consequence of chronic BPS therapy. The exact etiology of BONJ is unknown. There has been some focus on chemical messengers for the arrested development of osteoblasts from stem cells, modulation of calcification, and inhibition of osteoclast action. According to the literature, the downregulation of the adhesive genes integrin aVb3 and tenascin C, which possibly even enhance the antiadhesive effect by autocrine secretion, could be one of the molecular, intracellular reasons for the BONJ. This effect of BPs could possibly explain the interindividual variability of BONJ incidence [75].

It follows from the above that BPs are a treatment of choice in PDB. The use of some drugs required for PDB treatment may be contraindicated in cases of marked renal impairment. In this case, the main route of drug clearance involves both glomerular filtration and proximal tubular secretion [76]. Therefore, CKD patients may exhibit impaired bioelimination and consequent accumulation of BPs, especially if administrated intravenously. This suggests that adverse effects involving the kidneys may be related to the maximal concentration rather than the area under the concentration–time curve. The adverse renal effects of intravenous BPs manifest themselves as glomerular sclerosis or acute tubular necrosis and are revealed by increases in serum creatinine concentrations. Due to their potential nephrotoxicity, BPs are generally contraindicated when GFR is lower than 30 mL/min/1.73 m^2^ [77]. Additionally, due to impaired renal function, living kidney donors can be at increased risk of acute kidney injury (AKI) caused by non-steroidal anti-inflammatory drugs (NSAIDs), commonly used to relieve pain that can be a manifestation of PDB [78,79]. It has been published that the use of analgesics is higher in PDB patients (average of 5.2 prescriptions for analgesics in PDB patients and 2.5 prescriptions in the group without PDB) [41]. In addition, the use of NSAIDs is associated with progressive loss of glomerular filtration rate in the onset of CKD, along with electrolyte disturbances and hypervolemia, conditions that may worsen the coexistent morbidities such as heart failure or hypertension [80]. NSAIDs that inhibit cyclooxygenase (COX) may adversely affect renal outcomes if used with other agents impacting glomerular hemodynamics (e.g., renin-angiotensin system inhibitors) or diuretics [81]. Due to the above-mentioned reasons, PDB with required antiPagetic therapy may be additional risk factors for chronic kidney disease after living donation.

### 3.7. Discussions on Kidney Donation from a Donor Suffering from PDB

Asymptomatic PDB does not have any special diagnostic markers apart from a high to very high activity of ALP, repeated in several measurements. In these cases, if the liver function is normal and other breakthrough bone conditions with high ALP activity are excluded, we would suggest performing a bone scintigraphy first. X-rays of the skull and facial bones, then abdomen and tibia (as proposed by Ralston [63]) result together in exposure to radiation almost comparable to a bone scan. The other advantage of bone scan is that it provides information about the polyostotic form during one examination (whole-body scan), and polyostotic form is often associated with complications [36]. Although the bone scan does not seem specific, an experienced nuclear physician is able to diagnose PDB based on some characteristic signs [55,56]. The advantage of X-ray examinations is that they are certainly easier to access when not in a larger center of reference or university.

Symptomatic PDB means pathological fractures, bone deformities, and “bodily pain” (bone pain). In symptomatic patients, diagnosis is usually made before qualifying for kidney donation. A problem for related kidney donors and recipients may be that PDB may be present as a familial disease. Approximately 15 to 40% of patients with PDB have an affected first-degree relative supporting genetic factors as contributory in affected individuals [82]. Current evidence suggests that the disease is genetically heterogeneous and can result from mutations in several different genes. The potential loci susceptibility to PDB is the PDB3 locus on chromosome 5q35, particularly mutations affecting the sequestosome 1 (SQSTM1) gene. Mutations of this gene were found in 20 to 50% of familial cases and 5 to 10% of sporadic cases of PDB [83].

It seems to us that we should refuse symptomatic kidney donors with PDB. Persons with asymptomatic (painless) PDB who are diagnosed with high serum ALP activity and polyostotic form visible in bone scan during the preparation for donation should be considered carefully and individually. Due to the possible complications of PDB and their treatment, we tend to exclude them from donation.

## 4. Conclusions

The risk–benefit analysis is important for assessing living kidney donor candidates. In any chronic condition revealed during the qualification process, a review of the current literature is crucial. Based on the potential therapeutic options as well as the possible complications of the underlying disorder, the decision must consider the guidelines and current medical knowledge in accordance with evidence-based medical practice. Local legal regulations must be followed. According to the opinion of the local Living Kidney Donation Program Qualification Board, a candidate for living kidney donation who suffers only from a monostotic and asymptomatic form of PDB can be considered as a living kidney donor. However, the patient assessed in the authors’ center diagnosed with monostotic but symptomatic PDB, with comorbidities (mild hypertension, nonalcoholic fatty liver disease, overweight), and leading a sedentary lifestyle was not a suitable candidate for living kidney donation and was withdrawn.

## Figures and Tables

**Table 1:** Pre-donation and post-donation risk prediction tools (the dots indicate the variable or result is included in the tool).

Authors	Outcomes				Predonation Variables												
	Predonation ESRD	Postdonation ESRD	Postdonation eGFR	Postdonation Proteinuria	Age	Gender	Race	Recipient Relation	Diabetes in Donor	Diabetes in Recipient	eGFR	Blood Pressure	Hypertension Meds	BMI	Smoking	UACR	Glycemia
Grams et al. [22]	•				•	•	•		•		•	•	•	•	•	•	
Ibrahim et al. [23]		•	•	•	•	•		•		•	•	•		•	•		•
Massie et al. [24]		•			•	•	•	•						•			

## Data Availability

Data sharing is not applicable to this article, as no datasets were generated or analyzed during the current study.
